# High Occurrence of Dementia in Older Adults Returning to Community From Prison

**DOI:** 10.1093/geroni/igaa057.843

**Published:** 2020-12-16

**Authors:** Amy Byers, Yixia Li, Lisa Barry

**Affiliations:** 1 University of California, San Francisco / San Francisco VA Health Care System, San Francisco, California, United States; 2 Northern California Institute for Research and Education, San Francisco, California, United States; 3 UConn Center on Aging, Farmington, Connecticut, United States

## Abstract

Very little is known about Alzheimer’s Disease and related dementias (AD/ADRD) and diseases (e.g., Parkinson’s/PD) in older adults returning to community from prison (“older reentry adults”). We utilized a national sample of older veterans to conduct the first study documenting the occurrence (prevalence/incidence) of AD/ADRD and related diseases in older reentry adults. We examined 28,235 reentry veterans who were aged 50 years and older at their most recent release date and Medicare beneficiaries, 2008 through December 31, 2017. AD/ADRD and related diseases were identified by ICD-9/10 codes in the electronic health record from Medicare and inpatient/outpatient Veterans Health Administration data. We examined distributions of AD/ADRD and related diseases across 4 age categories (sample %): 50-64 (55%), 65-74 (37%), 75-84 (7%), and 85+ (0.7%). Of the 28,235 veterans 50 years and older, 17% (n=4,725) had dementia (defined by AD/ADRD based on NIA criteria) or mild cognitive impairment (MCI) and 3% (n=794) had PD. Of those with dementia/MCI, 18% had AD and 26% MCI. Nearly 40% of dementia diagnoses occurred prior to/on most recent release date, and 40% of PD diagnoses occurred prior to/on most recent release date. Differences were significant across age groups (P<.001), with very high rates of diagnoses across all age groups, as well as indicative of high occurrence of early onset dementia and “accelerated aging” [50-64, 14%; 65-74, 18%; 75-84, 33%; and 85+, 53%]. This study is a first step in filling a major research gap by describing AD/ADRD, MCI and related diseases in reentry adults.

